# The role of loneliness and learning burnout in the regulation of physical exercise on internet addiction in Chinese college students

**DOI:** 10.1186/s12889-023-16783-5

**Published:** 2023-10-12

**Authors:** Kun Wang, Yan Li, Yi Yang, Tingran Zhang, Jiong Luo

**Affiliations:** 1 College of Physical Education, Research Centre for Exercise Detoxification, Southwest University, NO.2 Tiansheng Road, Beibei District, Chongqing, 400715 China; 2 College of Liberal Studies, Chongqing Industry Polytechnic College (Sports Work Department), Yubei, Chongqing, 401120 China

**Keywords:** College students, Internet addiction, Physical exercise, Loneliness, Learning burnout, Chain mediation

## Abstract

**Objective:**

To explore the mediating effects of physical exercise on internet addiction in Chinese college students and to reveal the mediating effects of loneliness and learning burnout on physical exercise and internet addiction.

**Methods:**

1238 Chinese college students were investigated by physical exercise scale, loneliness scale, learning burnout scale, and internet addiction scale. The SPSS 27.0 was used to conduct a correlation analysis of the data and AMOS21.0 was used to establish a structural equation model to investigate the mediation effect.

**Results:**

Physical exercise could directly negatively predict the internet addiction of college students. Loneliness and learning burnout partially mediate the relationship between physical exercise and internet addiction, and the mediating pathways included “physical exercise-loneliness-internet addiction”, “physical exercise-learning burnout-internet addiction”, and “physical exercise-loneliness-learning burnout-internet addiction”, accounting for 9.38%, 15.63% and 21.88% of the total effect, respectively. In the chain mediation effect, it was mainly the loneliness and sub-dimensions of learning burnout (low mood, misbehavior) that were at play.

**Conclusion:**

Physical exercise not only directly affects internet addiction of college students but also indirectly affects internet addiction through the independent mediating effect of loneliness and learning burnout and the chain mediating effect of “loneliness-learning burnout”.

## Introduction

The 50th Statistical Report on the Development of the Internet in China shows that by June 2022, the number of internet users in China has reached 1.051 billion, and the internet penetration rate was as high as 74.4%. Among them, teenagers were the most active group of netizens, especially college students, the internet has penetrated their daily study and life and becomes an indispensable part [1]. However, the internet is undoubtedly a “double-edged sword”, because it allows college students to experience and enjoy its richness and convenience, but also easily makes them fall into the vortex of “Internet addiction”. Internet addiction refers to a kind of mental behavior disorder caused by repeated excessive use of the internet by individuals, which is manifested as a strong desire and increased tolerance for the reuse of the internet, withdrawal reaction when stopping or reducing the use of the internet, and may be accompanied by mental and physical pathological symptoms [2, 3], such as compulsive internet behavior, internet addiction withdrawal reaction, internet addiction tolerance, interpersonal and health problems, and time management problems [4]. The research points out that internet addiction will have a serious negative influence and harm Chinese college students’ studies, interpersonal relationships, and physical and mental health [5]. Therefore, in the increasingly severe situation of internet addiction among college students, it is of great significance to explore the influencing factors and mechanisms of internet addiction for the prevention or improvement of internet addiction among college students in China and even around the world.

### Physical exercise and internet addiction

As a green, safe, and environmentally friendly intervention strategy, physical exercise has been proven to have a positive effect on preventing or improving individual internet addiction [6]. For example, adolescents who regularly participate in physical activity tend to have higher levels of physical and mental health and lower levels of Internet addiction [7], and high physical exercise can directly negatively predict the internet addiction of college students [8]. Studies have found that long-term Tai Chi exercise could alleviate the level of internet addiction of Chinese college students by reducing their scores of compulsive internet behavior, internet addiction withdrawal reaction, internet addiction tolerance, interpersonal and health problems, and time management problems [9]. The mechanism of physical exercise on internet addiction was relatively complex. Petronel [6] pointed out that participating in diversified physical activities could strengthen the connection between teenagers and their peers in real life, to help them get rid of the programmed and virtualized network social interaction environment. Meanwhile, physical exercise could improve the mental health level of internet addicts, which was manifested as relief of somatization symptoms, relief of anxiety, a catharsis of bad emotions, and establishment of positive coping styles [10]. It can be seen that regular physical exercise has different degrees of influence on college students’ internet addiction and its sub-dimensions. Therefore, this study proposed hypothesis H1 that physical exercise has a direct negative predictive effect on college students’ internet addiction and its sub-dimensions.

### The mediating effect of loneliness

College students are at an important stage of developing peer relationships, pursuing autonomy and individuation, and are susceptible to loneliness [11]. Loneliness refers to a kind of negative emotion caused by an individual’s desire for social interaction and the gap between them and the actual level [12], which was considered to be a significant predictor of internet addiction among college students [13], and college students with higher emotional experiences of loneliness were more likely to develop internet addiction [14]. The loneliness of college students has a predictive effect on their tendency to internet addiction, showing that self-loneliness and developmental loneliness can predict their tolerance and withdrawal reaction to compulsive internet use and internet addiction [15]. Davis pointed out in the cognitive-behavioral model [16] that individuals with higher feelings of loneliness were more inclined to overuse the internet as psychological compensation. Interestingly, physical exercise was believed to be able to effectively improve individual loneliness. Physical exercise contains an important social component, which helps to meet teenagers’ social needs and expectations, improve their interpersonal and social skills, and prevent loneliness [17, 18]. Further studies have found that exercise could improve and regulate the mental health of college students, release their psychological pressure to a certain extent, increase social opportunities through exercise, reduce negative emotions such as loneliness, and thus relieve mobile phone addiction [19]. Therefore, this study proposes hypothesis H2 that loneliness has a mediating effect between physical exercise and internet addiction and its sub-dimensions.

### The mediating effect of learning burnout

Learning burnout is an important cause of internet addiction among college students. It refers to the negative attitude and behavior of weariness of learning due to learning pressure or lack of interest, which is usually manifested as low mood, misbehavior, and low sense of achievement [20], as well as the lack of recognition of the necessity of learning or student identity. Learning burnout will not only reduce students’ academic performance but also increase the occurrence of truancy [21]. Studies showed that there was a significant positive correlation between learning burnout and internet addiction among college students [22], showing that low mood was positively correlated with internet addiction tolerance, interpersonal and health problems, and misbehavior was significantly positively correlated with interpersonal and health problems and time management problems [23]. The essence of internet addiction caused by learning burnout may be unsatisfied needs [24], and if college students cannot be satisfied with their studies, they are likely to turn to the internet to seek compensation, and eventually, it is easy to lead to internet addiction [25]. As a personal resource, physical exercise can reduce individual pressure and prevent burnout symptoms [26], enhance individual mental health, reduce fatigue and relieve tension, and facilitate the recovery of emotional resources, thus reducing learning burnout [27]. Some competitive and interesting exercise programs can effectively improve the sense of achievement of teenagers with internet addiction, thus reducing their degree of internet addiction [28]. Therefore, this study proposes hypothesis H3 that learning burnout has a mediating effect between physical exercise and internet addiction and its sub-dimensions.

### The chain mediation effects of loneliness and learning burnout

**Fig. 1:**
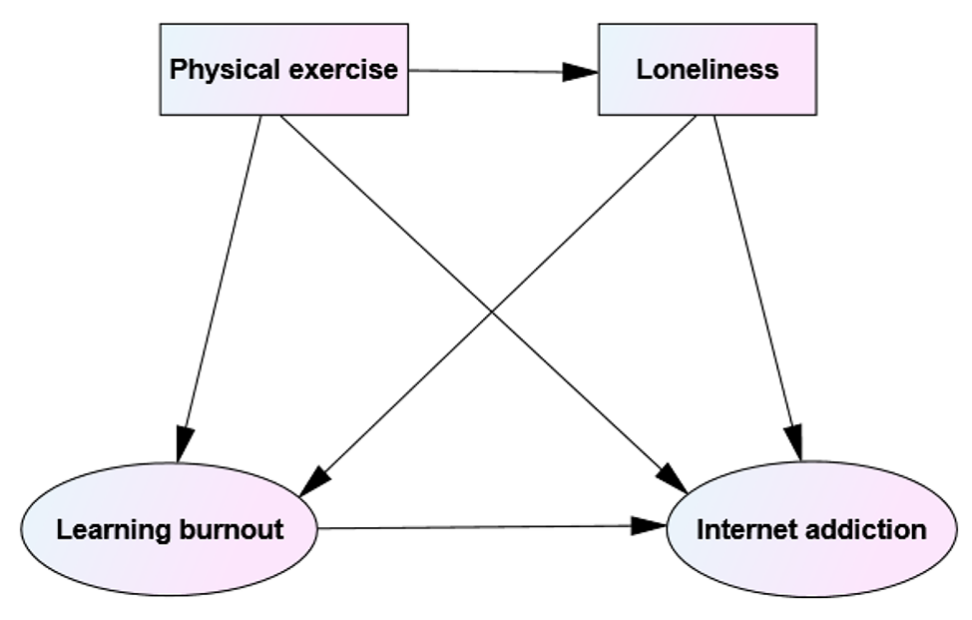
Hypothesis diagram of mediating model between loneliness and learning burnout in physical exercise and internet addiction

The individual-Emotion-cognitive-executive model (I-PACE) of internet addiction points out that individual characteristics will affect executive function and inhibitory control through emotional and cognitive characteristics, thus affecting internet addictive behaviors [29]. According to the model, physical exercise, a personal resource, may influence internet addictive behavior through the combination of emotional and cognitive characteristics such as loneliness and learning burnout. Although there are few studies on the correlation between loneliness and learning burnout, limited studies have shown that compared with college students with less loneliness, those with a higher degree of loneliness felt tired in the learning process and had a higher frequency of symptoms such as a low sense of achievement, interpersonal alienation, negative learning emotions, and emotional exhaustion [30]. However, few studies have explored the role of loneliness and learning burnout in the influence of physical exercise on the path of individual internet addiction at present, as well as the path relationship between the sub-dimensions of variables, especially Chinese college students who are prone to internet addiction. Therefore, based on the previous three research hypotheses, this study proposed hypothesis H4 for Chinese college students that loneliness and learning burnout have a chain mediation effect between physical exercise and internet addiction and its sub-dimensions (see Fig. 1 for the hypothesis diagram of the mediation model).

## Participants and methods

### Participants

This study adopts a cross-sectional research design to conduct a random sample survey of college students in three universities in Southwest China, and all participants (including freshmen to seniors) were voluntary participants. Firstly, to ensure the authenticity of the questionnaire filling, after the questionnaire content and precautions were fully explained to the participants, the survey was conducted in the form of on-site questionnaire distribution and supervision, and the time for participants to fill in the questionnaire should not be less than 10 min. Secondly, after the participants fill out the questionnaire, they need to hand it to the on-site staff to recover the questionnaire data. Finally, the staff summarized all the completed questionnaires and found that a total of 1500 questionnaires were issued in this study, and 1342 were recovered, with a recovery rate of 89.47%. In addition, to improve the accuracy of the data, 104 samples with unknown key information, incomplete filling, random filling, and missing data were eliminated in the data pre-processing through descriptive statistics, numerical conversion, and missing value processing, and 1238 valid questionnaires were obtained, with an effective rate of 92.25%. Demographic data showed that the average age of the participants was 19.96 ± 1.59 years, there were 689 males (55.65%) and 549 females (44.35%). Among them, were 480 freshmen (38.77%), 269 sophomores (21.73%), 278 juniors (22.46%), and 211 seniors (17.04%). It should be pointed out that the grade division was carried out following the unified standards of China. This study was approved by the Ethics Committee of the School of Physical Education, Southwest University of China (SWU-TY202105) and followed the Declaration of Helsinki, and written informed consent was obtained from all participants.

### Measurement tools

#### Internet addiction scale

The selected Internet Addiction Scale was compiled by Chen et al. [4]. There were 26 items on the scale, including 5 sub-dimensions: compulsive internet behavior, internet addiction withdrawal, internet addiction tolerance, interpersonal relationship and health, and time management. The Likert 4-point scoring was adopted, the total score of internet addiction was the sum of 20 items, and the score range was 26 to 104 points. The higher the score, the higher the level of addiction. In this study, the retest reliability of this scale was high, and the correlation coefficient r = 0.83. Each factor load was greater than 0.5, AVE was greater than 0.5, and the combined reliability CR was greater than 0.8, indicating that the scale has good convergent validity. Meanwhile, the Cronbach α coefficient of this scale was 0.91, and the coefficients α of the sub-dimensions were 0.87, 0.90, 0.90, 0.92, and 0.89, respectively. The results of confirmatory factor analysis were as follows: x^2^/df = 1.96, RMSEA = 0.04, AGFI = 0.98, TLI = 0.97, CFI = 0.99, IFI = 0.96, GFI = 0.99. It showed that the scale has good reliability and validity in this study.

#### Loneliness scale

The Loneliness Scale (UCLA) was compiled and revised by Russell et al. [12, 31], which has been translated and verified in Chinese college students [32]. There were 20 items on the scale, which was a single dimension. The Likert 4-point scoring was adopted, the total score of loneliness the sum of 20 items, and the score range was 20 to 80 points. The higher the score, the stronger the loneliness. In this study, the retest reliability of this scale was high, and the correlation coefficient r = 0.86. Factor load was greater than 0.5, AVE was greater than 0.5, and combined reliability CR was greater than 0.7, indicating that the scale has good convergent validity. Meanwhile, the Cronbach α coefficient was 0.80. It showed that the scale has good reliability and validity in this study.

#### Learning burnout scale

The selected Learning Burnout Scale was compiled by Lian et al. [20] for college students. The scale consists of 20 items, including 3 sub-dimensions: low mood, misbehavior, and low sense of accomplishment. The Likert 5-point scoring was adopted, the total score of learning burnout was composed of scores of 20 items, and the score range was 20 to 100 points. The higher the score, the higher the level of learning burnout. In this study, the retest reliability of this scale was high, and the correlation coefficient r = 0.78. Each factor load was greater than 0.7, AVE was greater than 0.6, and the combined reliability CR was greater than 0.7, indicating that the scale has good convergent validity. Meanwhile, the Cronbach α coefficient of the total table was 0.86, and the Cronbach α coefficients of the sub-dimensions were 0.86, 0.71, and 0.77, respectively. The results of confirmatory factor analysis were as follows: x^2^/df = 2.12, RMSEA = 0.06, AGFI = 0.93, TLI = 0.97, CFI = 0.92, IFI = 0.94, GFI = 0.96. It showed that the scale has good reliability and validity in this study.

#### Physical activity rating scale

The Physical Activity Rating Scale (PARS-3) compiled by Liang [33] was adopted to evaluate the physical exercise status of the subjects from the three aspects of exercise intensity, frequency, and time. The Likert 5-point scoring was adopted, and the exercise intensity and frequency from weak to strong were respectively calculated as 1 to 5 points, and exercise time from weak to strong was calculated as 0 to 4 points. The formula “exercise intensity × exercise time × exercise frequency” was used to quantify the total score of exercise behavior, with the score ranging from 0 to 100, and the higher the score, the greater the exercise amount. In this study, the retest reliability of this scale was high, and the correlation coefficient r = 0.82. Factor load was greater than 0.5, AVE was greater than 0.6, and combined reliability CR was greater than 0.5, indicating that the scale has good convergent validity. Meanwhile, the Cronbach α coefficient of this scale was 0.83. It showed that the scale has good reliability and validity in this study.

### Data

SPSS27.0 was used for data processing and analysis in this study. Firstly, descriptive analysis was used to make statistics on the sample data, and exploratory factor analysis and confirmatory factor analysis were used to test the reliability and validity of the scale. Secondly, the Pearson correlation analysis was used to investigate the correlation between variables. Finally, AMOS21.0 was used to establish the structural equation model and path model to investigate the mediating effects of loneliness and learning burnout on physical exercise and internet addiction and their sub-dimensions, and the Bootstrap mediation test was used to test the mediating effects. The significance level of all indicators was set at P < 0.05.

## Results

### Common method deviation test

During the test, this study adopted anonymous questionnaire measurement, positive and negative scoring, standardized test, and other program control methods to carry out control, and Harman single factor test was used to investigate the common method deviation [34]. The results showed that there were 10 factors with characteristic roots greater than 1, and the variance explained by the first factor was 31.18%, less than the critical standard of 40%. Therefore, there was no problem with common methodology bias.

### Correlation analysis of physical exercise, loneliness, learning burnout, and internet addiction

The results of the correlation analysis showed that (Table 1): physical exercise was negatively correlated with loneliness, learning burnout, and internet addiction. There was a significant positive correlation between loneliness and learning burnout and the five sub-dimensions of internet addiction, respectively. There was a significant positive correlation between learning burnout and five sub-dimensions of internet addiction. The correlation between the study variables was significant, which provides a good basis for the subsequent test of the mediation effect.

**Table 1:** Correlation coefficients among physical exercise, loneliness, learning burnout, and five sub-dimensions of internet addiction

Variables	PE	LL	LB	COB	IAWR	IAT	IRH	TM		
PE	1.00									
LL	-0.26^***^	1.00								
LB	-0.26^***^	0.53^***^	1.00							
COB	-0.27^***^	0.36^***^	0.50^***^	1.00						
IAWR	-0.26^***^	0.35^***^	0.46^***^	0.80^***^	1.00					
IAT	-0.29^***^	0.36^***^	0.44^***^	0.74^***^	0.79^***^	1.00				
IRH	-0.29^***^	0.40^***^	0.48^***^	0.70^***^	0.72^***^	0.75^***^	1.00			
TM	-0.26^***^	0.39^***^	0.49^***^	0.70^***^	0.72^***^	0.60^***^	0.83^***^	1.00		
Mean	24.15	43.44	56.28	10.96	10.49	8.63	14.58	10.75		
SD	25.41	7.80	10.58	3.24	3.21	2.66	4.42	3.35		

Note: ***means P < 0.001. PE means physical exercise, LL means loneliness, LB means learning burnout, COB means compulsive online behavior, IAWR means internet addiction withdrawal reaction, IAT means internet addiction tolerance, IRH means interpersonal relationship and health, and TM means time management

### The mediating effect of loneliness and learning burnout on physical exercise and internet addiction

**Fig. 2:**
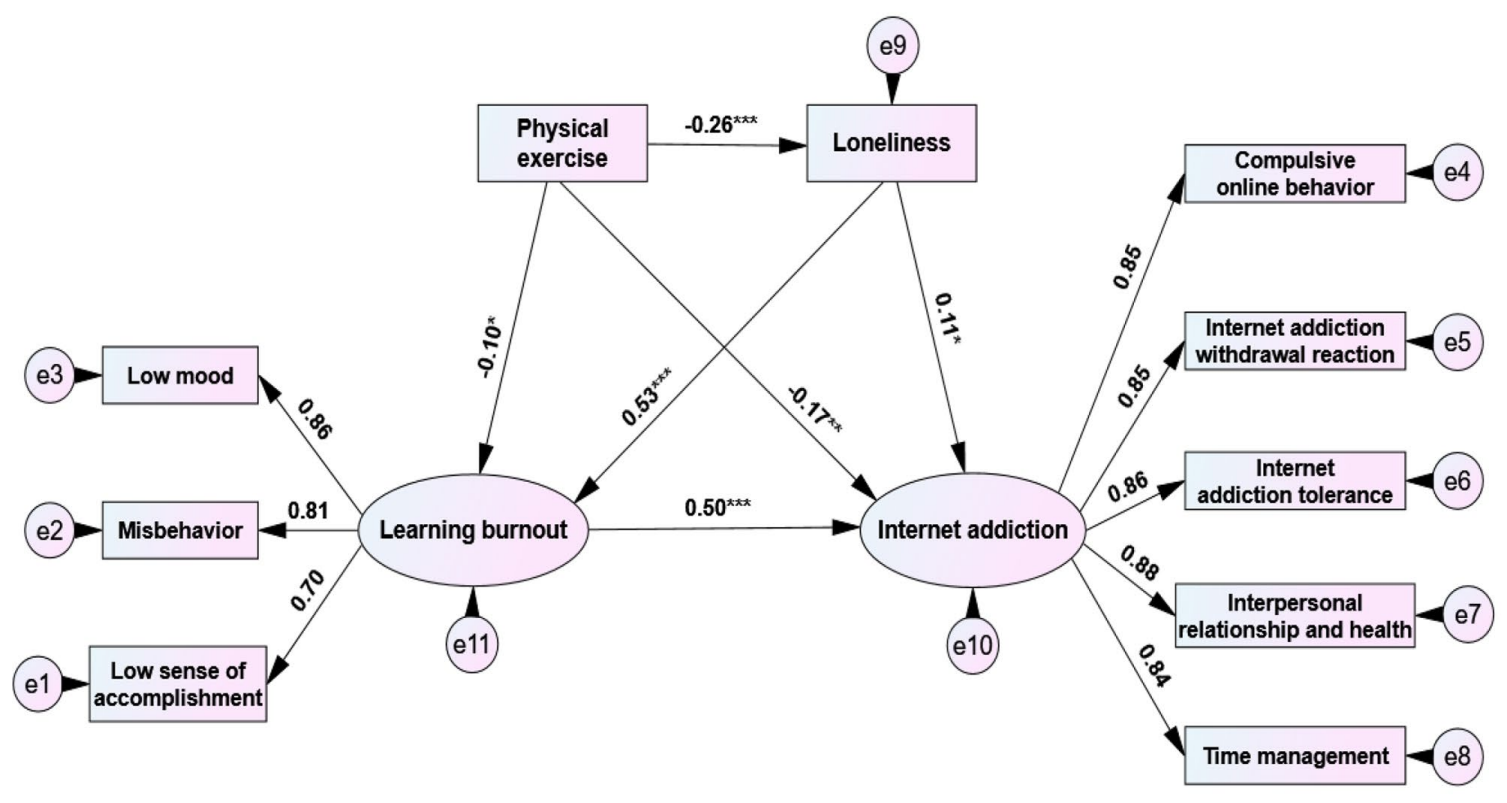
Chain mediation model of loneliness and learning burnout between physical exercise and internet addiction. Note:*means P < 0.05,**means P <0.01,***means P < 0.001.

According to the process of mediating effect test proposed by Wen et al. [35], this study investigated the path relationship between physical exercise, loneliness, learning burnout, and internet addiction among college students (Fig. 2). First, the total effect of physical exercise on internet addiction was tested, and then the model fitting and the significance of each path coefficient were tested after adding mediating variables (loneliness, learning burnout). Moreover, gender, age, and grade were controlled in the structural equation model in the form of covariables. In the total effect model, physical exercise directly and negatively predicted internet addiction (β1=-0.31, P < 0.001). After adding the two mediating variables of loneliness and learning burnout, the path coefficient of physical exercise on internet addiction decreased from β1 to β2, but the path coefficient still reached the significant level (β2=-0.17, P = 0.006), and the fitting indexes of the total effect and the mediating effect model reached the acceptable level.

The results of mediating effect test showed that: (1) Physical exercise could significantly negatively predict loneliness (β=-0.26, P < 0.001), and loneliness could significantly positively predict internet addiction (β = 0.11, P = 0.033), indicating that the mediating effect of “physical exercise → loneliness →internet addiction” was significant, and its effect size was − 0.26 × 0.11=-0.03. (2) Physical exercise could significantly negatively predict learning burnout (β=-0.10, P = 0.039), and learning burnout could significantly positively predict internet addiction (β = 0.50, P < 0.001), indicating that the mediating effect of “physical exercise → learning burnout →internet addiction” was significant, and its effect size was − 0.10 × 0.50=-0.05. Moreover, according to the research suggestion of Taylor et al. [36] on the probability and statistical efficacy of type I errors when there are “mediating effects of three paths”, the joint significance method was adopted to test the chain mediating effect of “loneliness →learning burnout” between physical exercise and internet addiction. The results showed that: (3) Loneliness could significantly positively predict learning burnout (β = 0.53, P < 0.001), indicating that the mediating effect of “physical exercise →loneliness →learning burnout →internet addiction” was significant, and the effect size was − 0.26 × 0.53 × 0.50= -0.07.

Through the effect decomposition of various paths between physical exercise and internet addiction (Table 2), it was found that the total effect of physical exercise on internet addiction was − 0.32, the ratio of direct effect to total effect was 53.13%, and the ratio of intermediary effect to total effect was 46.87%. Among them, the mediating effect of loneliness accounted for 9.38%, the mediating effect of learning burnout accounted for 15.63%, and the mediating effect of “loneliness →learning burnout” accounted for 21.88%, and the mediating effect size of the chain was significantly higher than that of the single one.

**Table 2:** Effect decomposition of physical exercise on internet addiction

Influence path	Standardized effect value	Total effect ratio	Boot 95%CI	Significance
Physical exercise →internet addiction	-0.17	53.13%	(-0.23, -0.15)	√
Physical exercise →loneliness →internet addiction	-0.26 × 0.11= -0.03	9.38%	(-0.07, -0.02)	√
Physical exercise →learning burnout →internet addiction	-0.10 × 0.50= -0.05	15.63%	(-0.09, -0.03)	√
Physical exercise →loneliness →learning burnout →internet addiction	-0.26 × 0.53 × 0.50= -0.07	21.88%	(-0.10, -0.06)	√
Total mediation effect	-0.15	46.87%	(-0.22, -0.11)	√
Total effect	-0.17 + (-0.15)= -0.32	100.00%	——	√

**Fig. 3:**
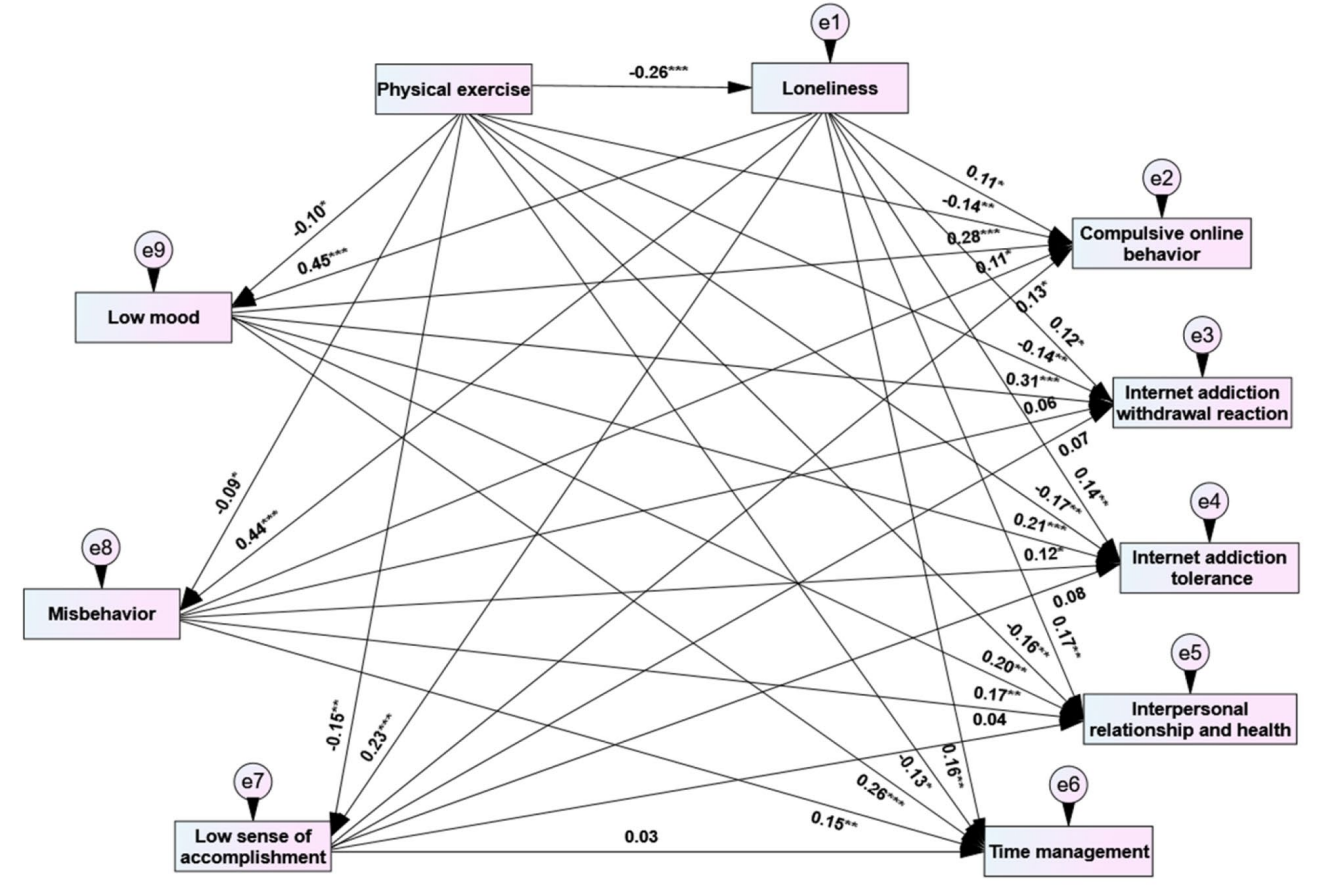
Path models of physical exercise, loneliness, learning burnout, and the sub-dimensions of internet addiction. Note:*means P <0.05, **means P <0.01, ***means P<0.001.

To further reveal the specific path mechanism of physical exercise affecting internet addiction, this study established a path relationship model among major variable quantum sub-dimensions (Fig. 3). The results showed that (Table 3): (1) “loneliness →low mood” had a significant chain mediating effect between the physical exercise and five sub-dimensions of internet addiction, and the effect values were − 0.03, -0.04, -0.02, -0.02, -0.03, respectively. (2) “loneliness →misbehavior” had significant chain mediating effects on physical exercise and compulsive internet behavior, internet addiction tolerance, interpersonal relationship and health, and time management, and the effect values were − 0.01, -0.01, -0.02, and − 0.02, respectively. (3) “loneliness →low sense of accomplishment” only had a significant chain mediating effect between physical exercise and compulsive online behavior, and the effect value was − 0.01. Therefore, all the hypotheses of this study have been effectively confirmed.

**Table 3:** Chain effect decomposition of physical exercise on sub-dimensions of internet addiction

Model	Influence path	Standardized effect value	Boot 95%CI	Significance
Model 1	PE→LL→ LM →COB	-0.26 × 0.45 × 0.28= -0.03	(-0.09, -0.02)	√
	PE→LL →LM→IAWR	-0.26 × 0.45 × 0.31= -0.04	(-0.07, -0.02)	√
	PE→LL →LM →IAT	-0.26 × 0.45 × 0.21= -0.02	(-0.03, -0.01)	√
	PE →LL →LM →IRH	-0.26 × 0.45 × 0.20= -0.02	(-0.04, -0.01)	√
	PE →LL→LM →TM	-0.26 × 0.45 × 0.26= -0.03	(-0.06, -0.02)	√
Model 2	PE →LL → MB → COB	-0.26 × 0.44 × 0.11= -0.01	(-0.05, -0.01)	√
	PE→LL → MB → IAWR	-0.26 × 0.44 × 0.06= -0.007	(-0.02, 0.02)	×
	PE →LL → MB → IAT	-0.26 × 0.44 × 0.12= -0.01	(-0.03, -0.01)	√
	PE →LL → MB → IRH	-0.26 × 0.44 × 0.17= -0.02	(-0.05, -0.01)	√
	PE→LL → MB → TM	-0.26 × 0.44 × 0.15= -0.02	(-0.04, -0.01)	√
Model 3	PE →LL → LSA → COB	-0.26 × 0.23 × 0.13= -0.01	(-0.04, -0.01)	√
	PE →LL → LSA → IAWR	-0.26 × 0.23 × 0.07= -0.004	(-0.02, 0.03)	×
	PE →LL → LSA → IAT	-0.26 × 0.23 × 0.08= -0.005	(-0.02, 0.01)	×
	PE→LL → LSA → IRH	-0.26 × 0.23 × 0.04= -0.002	(-0.01, 0.02)	×
	PE →LL → LSA → TM	-0.26 × 0.23 × 0.03= -0.002	(-0.02, 0.02)	×

Note: PE means physical exercise, LL means loneliness, LM means low mood, MB means misbehavior, LSA means a low sense of accomplishment, COB means compulsive online behavior, IAWR means internet addiction withdrawal reaction, IAT means internet addiction tolerance, IRH means interpersonal relationship and health, TM means time management

## Discussion

### The direct effect of physical exercise on internet addiction

This study found that physical exercise has a significant direct negative predictive effect on internet addiction among college students. A study has shown that physical exercise could regulate the relationship between internet addiction and adolescents’ psychological and physical symptoms [37]. Meanwhile, the previous study only generally stated that physical exercise has positive effects on reducing individual internet addiction [7], and did not investigate the effect relationship between variables from the dimension level. This study found that physical exercise has a direct negative predictive effect on compulsive internet behavior, internet addiction withdrawal reaction, internet addiction tolerance, interpersonal relationship and health, and time management of college students. This was more consistent with previous studies [9], indicating that college students could satisfy their inner needs through physical exercise instead of using the internet, to prevent or improve internet addiction from psychological and behavioral levels. The endogenous mechanism could be explained as that physical exercise improves the exercisers’ excitement and pleasant experience, and when the pleasant emotional experience was generated, it will induce positive emotions and the desire to try again, and could properly offset the influence of depression and anxiety to improve the psychological state [38]. Meanwhile, regular participation in physical exercise could effectively shorten the time and energy college students invest in the internet, avoid excessive use of the internet, and the corresponding addictive behavior.

### The mediating role of loneliness and learning burnout between physical exercise and internet addiction

In addition to the direct effect, this study also found that loneliness has a partial mediating effect between physical exercise and internet addiction. College students are in the stage of role transformation and adaptation, they need to adapt to or construct new interpersonal relationships in the new environment, which is easy to cause trouble and psychological disorders [11], and thus prone to emotional experiences such as loneliness. Among them, excessive use or dependence on the internet was considered to be a common deviant behavior of lonely people, and loneliness has been confirmed to be significantly positively correlated with the pathological internet use of college students [13], which could predict the tolerance and withdrawal reaction of compulsive internet use and internet addiction [39]. According to the cognitive-behavioral model, individuals with higher feelings of loneliness were usually more inclined to overuse the internet as psychological compensation [16], to escape from reality and vent emotions. Adolescents with high loneliness could use the internet to make up for the lack of social cognition in reality or to escape the pain of loneliness, anxiety, and other negative emotions [40]. Interestingly, participation in physical exercise could significantly improve the sense of belonging and interpersonal skills of adolescents with internet addiction, thus improving their degree of internet addiction [18]. This study suggested that the experience of loneliness could be reduced by physical exercise, and college students with low loneliness were more willing to socialize or participate in collective activities, which was conducive to improving their compulsive internet behavior, internet addiction withdrawal reaction, internet addiction tolerance, interpersonal and health problems, and time management problems.

Secondly, learning burnout plays a partial mediating role between physical exercise and internet addiction. In the face of unknown fields, college students are prone to frustration, low sense of achievement, lack of learning control, and increased time pressure will aggravate the sense of learning burnout of college students [41]. Learning burnout has a positive predictive effect on internet addiction, and low mood and misbehavior in learning burnout have a positive predictive effect on internet addiction [42], while physical exercise has been proved to significantly reduce the learning burnout of college students and has a negative direct predictive effect on learning burnout [43], and could prevent learning burnout by reducing an individual’s physical vulnerability to stress [44]. It should be noted that reducing students’ academic pressure has an inhibitory effect on internet addiction to some extent [25]. The cognitive-behavioral model holds that pathological internet behaviors will be affected by adverse tendencies and stressors, and the psychological basis of internet addiction among college students was the non-adaptive cognition formed on the internet, which further strengthens their behaviors of internet obsession [16]. Once college students have depression, burnout, and other emotions in learning, they are more likely to use online games to cover up and avoid the pain in reality, make up for the sense of loss, and seek the need for achievement and a sense of value [45]. Moreover, low mood could effectively predict compulsive internet use, internet addiction withdrawal reaction and interpersonal and health problems of online game addicts, and misbehavior could effectively predict internet addiction tolerance [45]. On this basis, this study found that the higher the amount of physical exercise, the lower the level of learning burnout, accompanied by a decline in compulsive online behavior, internet addiction withdrawal reaction, internet addiction tolerance, interpersonal and health problems, and time management, ultimately conducive to the prevention or improvement of internet addiction degree of college students, improve the level of mental health.

Finally, physical exercise may indirectly affect internet addiction through the chain mediating effects of loneliness and learning burnout. Physical exercise was considered a personal resource [26], which has positive effects on reducing loneliness and other emotional characteristics, releasing pressure, and preventing or relieving learning burnout [26, 38]. Meanwhile, the I-PACE model proposed by Brand et al. [29] carries out attribution analysis on internet addiction behavior from the logic chain of individual-emotion-cognition-execution. Through the chain mediation model, this study found that the chain mediation effect of loneliness and learning burnout was valid, and the effect size of the chain mediation was higher than that of loneliness or learning burnout alone. This may be related to the fact that loneliness exacerbates some of the difficulties college students face in adapting to college life, resulting in them feeling too tired to study [30]. Further through the path model, it was found that the chain mediation path of “physical exercise → loneliness → learning burnout → internet addiction” was relatively complex. It showed that physical exercise could significantly negatively predict loneliness, and loneliness could positively predict depression and misbehavior, and finally have significant predictive power on compulsive internet behavior, internet addiction withdrawal reaction, internet addiction tolerance, interpersonal relationship and health, and time management. In this process, misbehavior did not significantly predict the withdrawal reaction of internet addiction, and a low sense of accomplishment only had a significant predictive power for compulsive online behavior. In short, regular participation in physical exercise could alleviate loneliness by increasing interpersonal communication and pleasant experience for college students, thereby promoting the release of bad emotions and satisfying realistic needs, reducing misbehavior, and ultimately helping to prevent the occurrence of excessive use of the internet behaviors or improve the negative emotions, interpersonal communication barriers and unreasonable time allocation caused by internet addiction.

### Limitations

Firstly, the cross-sectional study design adopted in this study makes it difficult to accurately infer the causal relationship between variables. In the future, cross-hysteresis or experimental study design can be used to test and improve the corresponding research results. Secondly, in terms of the consideration and selection of mediating variables, this study only examined the chain mediating effect of loneliness and learning burnout, while the path of physical exercise affecting internet addiction may involve more mediating or moderating variables, which can be further discussed in the future. Finally, this study only conducted a sample survey of some college students in southwest China, and the sample has certain limitations. In the future, more regions can be included for large sample comparison and tracking research. Meanwhile, whether the prediction model established in this study is consistent with other populations (such as children) needs further verification.

## Conclusions

Physical exercise can not only negatively affect the internet addiction of college students directly, but also indirectly affect the internet addiction through part mediating effects of loneliness and learning burnout respectively. On this basis, this study also found that physical exercise could prevent or improve the internet addiction of college students through the chain mediation effect of loneliness and learning burnout. Among them, in the chain mediation pathway relationship between physical exercise and internet addiction and its sub-dimensions, loneliness and two sub-dimensions of learning burnout (low mood and improper behavior) were mainly playing a role.

## Data Availability

The datasets generated and/or analysed during the current study are not publicly available due privacy but are available from the corresponding author on reasonable request.

## References

[1] Wang XJ, Gao SQ, Liu S (2022). Impact of social exclusion on college students’ internet addiction: mediation of self-concept clarity. China J Health Psychol.

[2] Tao R, Wang JL, Huang XQ, Liu CY, Yao SM, Xiao LJ (2008). Nomenclature, definition and clinical diagnostic criteria of internet addiction. Med J Chin People’s Armed Police Force.

[3] Spada MM (2014). An overview of problematic internet use. Addict Behav.

[4] Chen SH, Weng LZ, Su YR (2003). The development of Chinese internet addiction scale and the study of psychometric characteristics. Chin J Psychol.

[5] Li T, Zhang LJ (2004). How college students’ internet addiction are related to parental rearing patterns. J Psychol Sci.

[6] Petronel CM (2014). The role of physical education has social integration of children dominated computer. Procedia-Social and Behavioral Sciences.

[7] Khan MA, Shabbir F, Rajput TA (2017). Effect of gender and physical activity on internet addiction in medical students. Pakistan J Med Sci.

[8] Du ZH, Zhang XL (2022). Analysis of the mediating effects of self-efficacy and self-control between physical activity and internet addiction among Chinese college students. Front Psychol.

[9] Yang CY, Zeng GF (2017). Influence of Taijiquan exercise on internet addiction of college students. Chin J School Health.

[10] Liu YH, Shi Y (2014). Case study on sports intervention to youth internet addiction. Sports & Science.

[11] Laursen B, Hartl AC (2013). Understanding loneliness during adolescence: developmental changes that increase the risk of perceived social isolation. J Adolesc.

[12] Russell D, Peplau LA, Ferguson ML (1978). Developing a measure of loneliness. J Pers Assess.

[13] Bozoglan B, Demirer V, Sahin I (2013). Loneliness, self-esteem, and life satisfaction as predictors of internet addiction: a cross-sectional study among turkish university students. Scand J Psychol.

[14] Yao MZ, Zhong ZJ (2014). Loneliness, social contacts and internet addiction: a cross-lagged panel study. Comput Hum Behav.

[15] Wang B. A study on the relationship between the loneliness and internet addiction tendency of college students. J Psychol Sci. 2006;061425–7. 10.16719/j.cnki.1671-6981.2006.06.034

[16] Davis RA (2001). A cognitive-behavioral model of pathological internet use (PIU). Comput Hum Behav.

[17] Haugen T, Safvenbom R, Ommundsen Y (2013). Sport participation and loneliness in adolescents: the mediating role of perceived social competence. Curr Psychol.

[18] Liu YH (2013). Experimental study on sports intervention of youth internet addiction. J Tianjin Univ Sport.

[19] Cheng PF, Zhang Z, Liu SL, Wang KX, Yang C, Li YH (2020). Study on psychological factors and exercise intervention of mobile phone addiction of college students. J Changsha Aeronaut Vocat Tech Coll.

[20] Lian R, Yang LX, Wu LH. Relationship between professional commitment and learning burnout of undergraduates and scales developing. Acta Physiol Sinica. 2005;(05):632–6.

[21] Bask M, Salmela-Aro K (2013). Burned out to drop out: exploring the relationship between school burnout and school dropout. Eur J Psychol Educ.

[22] Wan HY, Yu JQ, Yan NQ, Huang JH (2021). Relationships between learning burnout and internet addiction among undergraduates during the novel coronavirus pneumonia: mediating effect of career adaptability. China J Health Psychol.

[23] Wei P, Yang S, Yu HB. Relationship between internet addiction disorder and learning burnout of college students. Chin J Clin Psychol. 2007;06650–1. 10.16128/j. cnki.1005-3611.2007.06.036.

[24] Zhang YL, Zhou ZY, Liu YJ, Xin SF (2022). The impact of boredom proneness on adolescents’ internet addiction: a moderated mediation model. Stud Psychol Behav.

[25] Zhang JJ, Chen H (2022). A follow-up study on academic pressure and internet addiction of college students in Jiangsu. Chin J School Health.

[26] Tang L, Zhang F, Yin R, Fan ZY (2021). Effect of interventions on learning burnout: a systematic review and meta-analysis. Front Psychol.

[27] Zhang ZY (2011). Study on the relationship between learning burnout, personality tenacity, and physical exercise. China Adult Education.

[28] Ma PS, Xia ZL, Li L, Liu Y (2022). Mechanisms, effects and strategies of exercise intervention on adolescents with internet addiction. J Shenyang Sport Univ.

[29] Brand M, Wegmann E, Stark R, Muller A, Wolfling K, Robbins TW (2019). The Interaction of person-affect-cognition-execution (I-PACE) model for addictive behaviors: Update, generalization to addictive behaviors beyond internet-use disorders, and specification of the process character of addictive behaviors. Neurosci Biobehavioral Reviews.

[30] Lin SH, Huang YC (2012). Investigating the relationships between loneliness and learning burnout. Act Learn High Educ.

[31] Russell DW (1996). UCLA Loneliness Scale (Version 3): reliability, validity, and factor structure. J Pers Assess.

[32] Wang YK, Lu Y, Chen JW (2023). Relationship between loneliness and mobile phone addiction in college students. Chin Mental Health J.

[33] Liang DQ. Stress level of college students and its relationship with physical activity. Chin Mental Health J. 1994; (1):5–6.

[34] Podsakoff PM, Mackenzie SB, Lee JY, Podsakoff NP (2003). Common method biases in behavioral research: a critical review of the literature and recommended remedies. J Appl Psychol.

[35] Wen ZL, Ye BJ (2014). Analyses of Mediating Effects: the development of methods and models. Adv Psychol Sci.

[36] Taylor AB, Mackinnon DP, Tein JY (2008). Tests of the three-path mediated effect. Organizational Res Methods.

[37] Lin L, Liu J, Cao X, Wen SY, Xu JC, Xue ZP (2020). Internet addiction mediates the association between cyber victimization and psychological and physical symptoms: moderation by physical exercise. BMC Psychiatry.

[38] Liu YH, Dan YJ (2009). A research on psychological attribution to internet addiction and intervention from exercise psychological perspective. J Beijing Sport Univ.

[39] Young K (1998). Caught in the net: how to recognize the signs of internet addiction-and a winning strategy for recovery.

[40] Sanders CE, Field TM, Diego M, Kaplan M (2000). The relationship of internet use to depression and social isolation among adolescents. Adolescence.

[41] Fan PY, Shang YH, Zhu B, Wang J, Guo CT, Jin JY (2021). Investigation and analysis of loneliness, learning burnout and resilience of medical students under the normalization of novel coronavirus pneumonia. China J Health Psychol.

[42] Wan HY, Yu JQ, Yan NQ, Huang JH. Relationships between learning burnout and internet addiction among undergraduates during the novel coronavirus pneumonia: mediating effect of career adapt ability. 2021;29(05):695–70110.13342/j.cnki.cjhp.2021.05.012.

[43] Li YS, Sun QL, Sun MZ, Sun PS, Sun QH, Xia X (2021). Physical exercise and psychological distress: the mediating roles of problematic mobile phone use and learning burnout among adolescents. Int J Environ Res Public Health.

[44] Khosravi M (2021). Burnout among Iranian medical students: prevalence and its relationship to personality dimensions and physical activity. Eur J Translational Myology.

[45] Wang B, Yu HB, Yang S (2007). The relationship between online game addiction and learning burnout of college students. Chin Mental Health J.

